# Comparative Evaluation of Silver Dioxide–Coated and Noncoated Stainless Steel Wires for Susceptibility to Corrosion: Protocol for an In Vitro Study

**DOI:** 10.2196/74309

**Published:** 2026-09-08

**Authors:** Aishwarya Atey, Rizwan Gilani, Shefali Singh, Abhijeet Kadam

**Affiliations:** 1 Department of Orthodontics and Dentofacial Orthopaedics, Sharad Pawar Dental College, Datta Meghe Institute of Medical Sciences, Orthodontics 102, 1st Floor, Sawangie (Meghe), Wardha, Maharashtra, 442001, India, 91 09284340255

**Keywords:** stainless steel arch wire (SS), corrosion resistance, silver dioxide coating, spectrometry, toxicity reduction

## Abstract

**Background:**

Stainless steel (SS) orthodontic archwires are widely used because of their mechanical properties, affordability, and biocompatibility; yet, prolonged intraoral exposure can cause electrochemical corrosion and release of metal ions (eg, Ni and Cr) that may affect clinical performance and patient safety. Surface coatings such as silver dioxide have been proposed to improve corrosion resistance, but comparative data for silver dioxide–coated versus uncoated SS orthodontic archwires across clinically relevant simulated oral environments are lacking.

**Objective:**

This study aimed to compare the corrosion susceptibility of silver dioxide–coated and noncoated 0.019“×0.025” SS orthodontic archwires in four simulated oral solutions by measuring electrochemical behavior, cumulative metal ion release, and mass lossthereby testing our null hypothesis (H₀) that no difference exists between coated and noncoated wires.

**Methods:**

This in vitro protocol uses 40 SS archwire specimens divided into two main groups (20 silver dioxide–coated, and 20 uncoated) and four immersion subgroups (n=5 per subgroup): (1) 0.9% NaCl, (2) phosphate-buffered solution acidified with lactic acid (pH ~6.75), (3) phosphate-buffered solution with 0.1 wt% NaF, and (4) phosphate-buffered solution with 0.1% w/v bovine serum albumin. Coatings will be deposited by radiofrequency magnetron sputtering to achieve a 15‐ to 20-nm silver oxide (Ag₂O/AgO) layer; coating quality will be verified through scanning electron microscopy (SEM), energy-dispersive x-ray spectroscopy (EDS), and profilometry. Specimens will be incubated individually at 37 °C with orbital agitation; immersion solutions will be sampled at baseline, 14 days, and 28 days. The primary outcome is corrosion current density (Icorr, µA/cm²) from potentiodynamic polarization. Secondary outcomes include pitting potential (Epit, mV vs SCE), cumulative ion release (Ag, Ni, Cr, Fe; expressed µg/mm²) analyzed by inductively coupled plasma–optical emission spectrometry (ICP-OES) after acidification, percent weight loss normalized to surface area (mg/cm²), and SEM/EDS surface analyses. Electrochemical tests will follow a standardized three-electrode setup (SCE reference). Statistical analyses will be used to assess normality; we will use independent-samples tests or nonparametric equivalents for between-group comparisons and mixed-effects models for time-dependent measures. Tests were evaluated at α=.05, with effect sizes and 95% CIs reported throughout.

**Results:**

Institutional ethical approval was obtained in April 2024 (DMIHER(DU)/IEC/2024/253). Coating parameters and quality control procedures have been established, and 40 specimens have been prepared and characterized. Immersion and electrochemical experiments are scheduled between June and December 2025, followed by ICP-OES analysis and statistical evaluation, with study completion planned by December 2025.

**Conclusions:**

This protocol will generate comparative data on electrochemical corrosion, ion release, and material degradation of silver dioxide–coated versus uncoated SS orthodontic archwires under multiple simulated oral conditions. Our findings will inform the potential of silver dioxide coatings to enhance the durability and biocompatibility of orthodontic archwires and will guide future in vivo or clinical investigations.

## Introduction

### Background

Orthodontic archwires remain in the oral cavity for prolonged durations, often ranging from 18 to 24 months, during which they are continuously exposed to a complex and dynamic intraoral environment. This prolonged exposure increases the likelihood of material degradation through electrochemical corrosion processes [1,2]. Stainless steel (SS) archwires have been widely used in orthodontics because of their favorable mechanical properties, corrosion resistance, affordability, and acceptable biocompatibility [3,4].

However, the oral environment is characterized by constant chemical fluctuations due to saliva composition, dietary intake, oral hygiene products, and fluoridated agents. These factors introduce electrolytes such as chloride ions, proteins, and fluoride, which can disrupt the passive oxide layer on metallic materials and initiate corrosion. Corrosion not only compromises the mechanical integrity of orthodontic appliances but may also result in the release of metal ions that affect surrounding oral tissues and systemic health [5,6].

The leaching of metallic ions from these materials has been linked to allergic reactions in some individuals. Orthodontic wires containing nickel are frequently used in treatment, but if they corrode and release nickel ions, they can trigger allergic responses in patients with nickel sensitivity [7,8]. The wet conditions in the mouth promote electrochemical corrosion, with metals generally remaining stable as long as their protective oxide layer remains intact [9,10]. However, SS has shown susceptibility to various types of corrosion such as pitting, stress, and crevice corrosion when exposed to chloride ions, amino acids, and proteins found in the oral environment [11,12]. When SS wires corrode, they can release metal ions that may be toxic to surrounding tissues [13-15].

Despite the wide use of SS in orthodontics, there is a lack of studies exploring the corrosion resistance of silver dioxide–coated and noncoated SS wires. This study aims to fill that gap by comparing the resistance to corrosion of silver dioxide–coated and noncoated SS orthodontic arch wires to understand their susceptibility to corrosion.

### Hypotheses

Our study hypotheses are as follows:

- Null (H₀): There is no significant difference in cumulative ion release or mass loss between silver dioxide–coated and noncoated wires.
- Alternative (H₁): Silver dioxide–coated wires have significantly lower cumulative ion release and mass loss than uncoated wires.

To our knowledge, this is the first in vitro protocol to systematically compare silver dioxide–coated SS orthodontic archwires with conventional archwires across multiple clinically relevant simulated oral environments.

### Aim

This study aims to evaluate and compare silver dioxide–coated and noncoated SS wires for susceptibility to corrosion.

## Methods

### Study Design

This study is designed as an in vitro experimental investigation and will be conducted at the Department of Orthodontics and Dentofacial Orthopedics, Sharad Pawar Dental College and Hospital, Wardha, Maharashtra, India.

### Sample Size Determination

The study aims to evaluate differences in corrosion susceptibility between silver dioxide–coated SS orthodontic archwires and noncoated SS archwires. As this investigation is intended as a pilot in vitro study, sample size estimation was informed by previously published electrochemical corrosion data. Based on mean differences and SD values of pitting potential (Epit) reported by Zhang et al [1], a minimum of five specimens per group per test condition was deemed sufficient to detect meaningful differences while maintaining feasibility. Accordingly, a total of 40 wire specimens were included in the study. This sample size is intended to support feasibility assessment and estimation of effect sizes for future adequately powered confirmatory studies.

### Sample Allocation and Grouping

Forty SS orthodontic archwire specimens were allocated into two main groups: silver dioxide– coated wires and uncoated control wires. Each group was further subdivided into four immersion subgroups corresponding to the simulated oral environments used in the study. Each subgroup consisted of five coated and five uncoated specimens, resulting in 10 specimens per solution and forty specimens overall.

### Silver Dioxide Coating Deposition

Silver dioxide coatings were deposited on SS archwires using radiofrequency magnetron sputter deposition. Deposition parameters, including base pressure, working pressure, argon and oxygen gas flow rates, radiofrequency power, and substrate temperature, were controlled and recorded for reproducibility. Oxide formation was achieved through reactive sputtering in an oxygen-containing atmosphere. The target coating thickness was maintained between 15 and 20 nm, as confirmed by surface profilometry and cross-sectional scanning electron microscopy (SEM).

### Quality Control and Coating Characterization

Coating integrity and uniformity were evaluated using SEM and energy-dispersive spectroscopy (EDS). Profilometry was used to verify coating thickness, and cross-sectional imaging was used to assess coating continuity. Adhesion of the coating was evaluated using a standardized tape adhesion test. At least three specimens from each coating batch were characterized to ensure consistency.

### Test Solutions and Experimental Conditions

Four simulated oral environments were prepared according to standardized protocols: modified Fusayama artificial saliva, a 0.9% sodium chloride solution, fluoridated artificial saliva supplemented with sodium fluoride, and protein-containing artificial saliva supplemented with bovine serum albumin (Multimedia Appendix 1). The pH of artificial saliva solutions was adjusted to 6.75 using lactic acid. All immersion tests were conducted at 37 °C to simulate intraoral temperature conditions.

### Immersion Testing

Each specimen was immersed individually in a sealed container containing a fixed volume of the designated test solution, maintaining a standardized surface area-to-volume ratio in accordance with ISO 10993‐12 guidelines. Immersion was carried out for 28 days, with intermediate solution collection at 14 days. After completion of the immersion period, specimens were cleaned using acetone, dried, and weighed using a precision balance to calculate percentage weight loss normalized to surface area.

### Electrochemical Corrosion Testing

Electrochemical testing was performed using a potentiostat-galvanostat system in a three-electrode configuration, consisting of a saturated calomel reference electrode and a platinum counter electrode. Specimens were embedded in epoxy resin, leaving a standardized exposed surface area. Prior to testing, samples were allowed to stabilize at open-circuit potential for 60 minutes. Potentiodynamic polarization and cyclic polarization tests were conducted to determine corrosion potential, corrosion current density (Icorr), pitting potential, and repassivation behavior.

### Ion Release Analysis

Immersion solutions collected at 14 and 28 days were acidified with nitric acid and analyzed using inductively coupled plasma–optical emission spectrometry (ICP-OES). The concentrations of silver, iron, nickel, and chromium ions released from the specimens were quantified and normalized to the specimen’s surface area.

### Surface Morphological Analysis

After immersion testing, specimens were examined using SEM to assess surface morphology and the presence of corrosion features such as pits. EDS was used for elemental mapping of corrosion products. Where feasible, pit number and distribution were quantified using image analysis software.

### Outcome Measures

The primary outcome measure was Icorr obtained from potentiodynamic polarization testing. Secondary outcome measures included pitting potential, metal ion release, percentage weight loss, and qualitative and quantitative surface characteristics observed through SEM and EDS analyses.

### Data Management and Statistical Analysis

All specimens were assigned coded identifiers to ensure traceability. Data were stored securely on institutional servers. Statistical analysis included assessment of data normality followed by appropriate parametric or nonparametric tests. A significance level of α=.05 was applied.

### Ethical Considerations

This study is an in vitro experimental investigation and does not involve human participants, biological tissues, or identifiable patient data. Institutional ethics approval was obtained from the Institutional Ethics Committee of Datta Meghe Institute of Higher Education and Research (DMIHER(DU)/IEC/2024/253).

## Results

Institutional ethics approval for this in vitro protocol was obtained from the Institutional Ethical Committee of Datta Meghe Institute of Higher Education and Research (DMIHER(DU)/IEC/2024/253) in April 2024. Standard operating procedures for silver dioxide coating, quality control characterization, and immersion solution preparation have been finalized.

The first batch of 40 SS archwire specimens (20 coated and 20 noncoated) has been prepared and characterized with respect to surface morphology, coating integrity, and baseline properties. These preparatory steps were completed between May and June 2024.

Immersion experiments in the four simulated oral solutions (chloride solution, artificial saliva, fluoridated artificial saliva, protein-containing artificial saliva) are scheduled to commence in June 2025 and are expected to be completed December 2025. Electrochemical corrosion testing will be conducted concurrently with immersion experiments.

**Figure 1.**
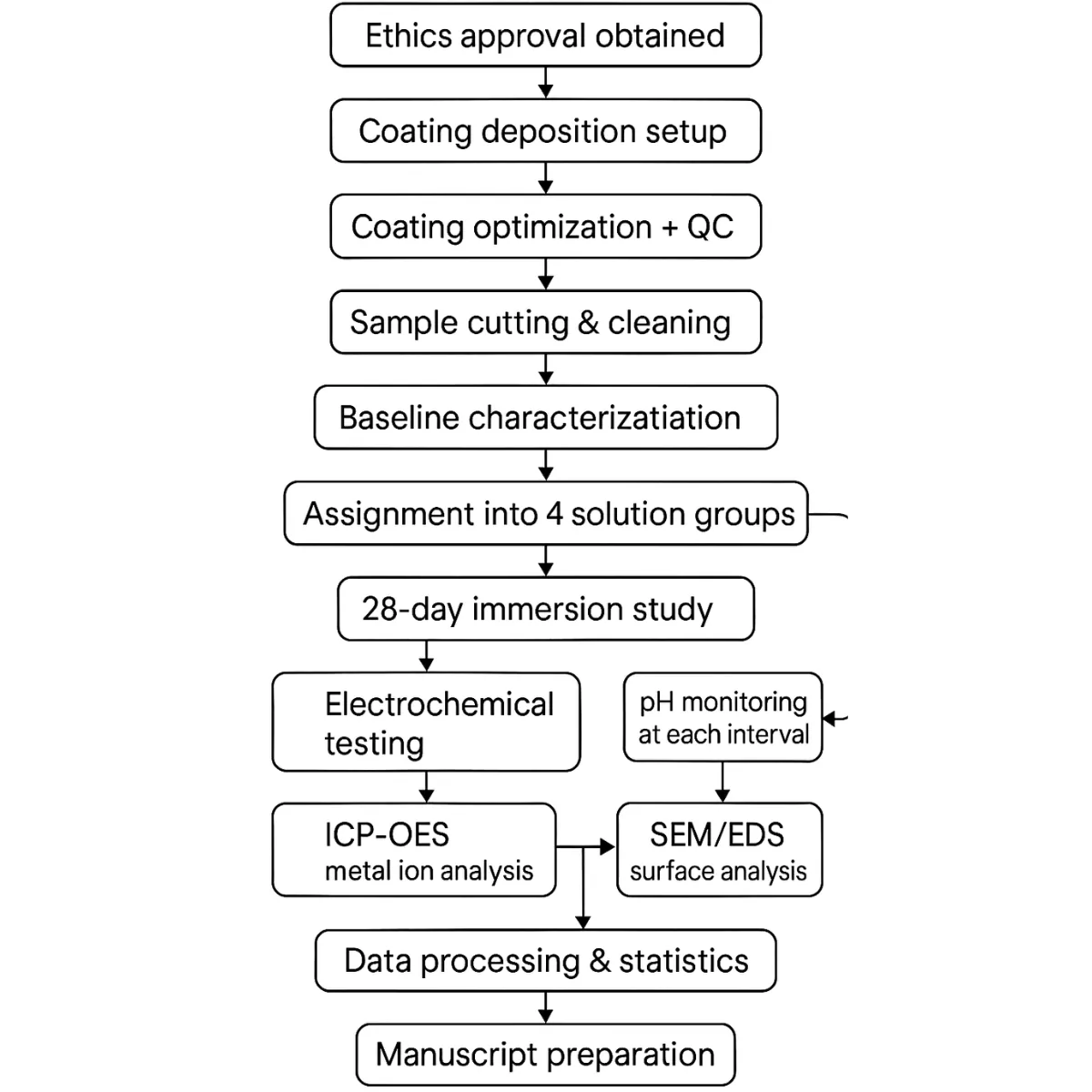
Schematic representation of the study workflow summarizing coating preparation, immersion protocol, analytical steps, and data management processes. EDS: energy-dispersive x-ray spectroscopy; ICP-OES: inductively coupled plasma–optical emission spectrometry; QC: quality control; SEM: scanning electron microscopy.

Subsequent ion release analysis using ICP-OES, surface characterization using SEM and EDS, and statistical data analysis are planned for completion between July and December. The overall study workflow and timeline are summarized in Figure 1. Dissemination of the study findings is planned through submission to peer-reviewed journals and presentation at scientific conferences.

## Discussion

### Principal Findings

This protocol is designed to systematically evaluate the corrosion susceptibility of silver dioxide–coated SS orthodontic archwire compared to that of conventional uncoated SS wire under simulated oral conditions. We anticipate that silver dioxide–coated wire will demonstrate lower Icorr, higher Epit, and reduced metal ion release across multiple immersion environments. These findings would support the hypothesis that surface modifications with silver dioxide may enhance the corrosion resistance and functional longevity of orthodontic archwire.

### Scientific Rationale and Interpretation

Orthodontic materials are continuously exposed to a complex and dynamic oral environment characterized by fluctuation pH, salivary enzymes, dietary components, and oral hygiene agents [16-19]. These conditions can compromise the passive oxide layer of metallic materials, leading to electrochemical, corrosion, and subsequent ion release. SS, although widely used owing to its favorable mechanical properties and cost-effectiveness, remains susceptible to localized corrosion in the presence of chloride ions, fluoride-containing agents, and organic components [20-24]. Surface modification strategies, such as silver dioxide coating, may provide a protective layer that stabilizes the surface oxide layer while also offering antimicrobial benefits [[25-30]].

### Comparison With Previous Studies

Previous studies in vitro have demonstrated that the corrosion behavior of orthodontic wires varies significantly, depending on the surrounding medium. Zhang et al [1] reported higher corrosion resistance in a protein-rich environment and increased susceptibility in chloride-containing solutions, highlighting the importance of evaluating material under multiple simulated oral conditions. Similarly, Gopikirsnan et al [2] observed differential ion release among orthodontic alloys, with certain materials exhibiting reduced nickel and chromium leaching, underscoring the clinical relevance of alloy selection and surface characteristic. The present protocol builds upon these findings by introducing silver dioxide surface modification and by using a multisolution experimental design to better simulate clinical variability.

### Strengths of the Study

A key strength of this protocol is its comprehensive evaluation of corrosion behavior using multiple complementary outcome measures, including electrochemical parameters, quantitative ion release analysis, surface characterization, and mass loss assessment. The inclusion of four distinct simulated oral environments enhances the ecological validity of these findings. Furthermore, the use of standardized coating characterization and a quality control procedure improves reproducibility and methodological rigor.

### Limitations

As an in vitro investigation, this study cannot fully replicate the complex biomechanical and biological conditions of the oral cavity, such as masticatory forces, thermal cycling, and biofilm-mediated corrosion. The absence of mechanical loading and microbial interactions may limit direct extrapolation of our findings to clinical settings. Additionally, the sample size is limited, and the study is intended as a pilot investigation rather than a definitive assessment of clinical performance. These limitations must be considered when interpreting our results.

### Future Directions

The findings generated from this pilot protocol are expected to provide preliminary evidence to inform larger-scale in vitro and future in vivo investigations. Incorporation of mechanical fatigue testing, thermal cycling, and biofilm models may further elucidate the long-term clinical performance of silver dioxide–coated orthodontic wires. Ultimately, such studies may contribute to the development of more corrosion-resistant and biocompatible orthodontic material.

### Dissemination Plan

The results generated from this protocol will be disseminated through publication in a peer-reviewed journal and presentation at national and international orthodontic and biomaterials conferences. Our findings will also inform future experimental designs and translational studies evaluating corrosion-resistant orthodontic material.

### Conclusion

The study may provide evidence that silver dioxide–coated SS archwires offer more favorable corrosion resistance in simulated oral environments, thus potentially proving their potential use in orthodontic treatments, which may suggest that they could be safer and a more durable option, contributing to better patient outcomes.

## Supplementary material

10.2196/74309 Multimedia Appendix 1 Coating parameters, solution recipes, and inductively coupled plasma–optical emission spectrometry analysis.

## Abbreviations

EDS: energy-dispersive x-ray spectroscopy
Epit: pitting potential
Icorr: corrosion current density
ICP-OES: inductively coupled plasma–optical emission spectrometry
SEM: scanning electron microscopy
SS: stainless steel
