# Supplementary material for: Comparative Evaluation of Silver Dioxide–Coated and Noncoated Stainless Steel Wires for Susceptibility to Corrosion: Protocol for an In Vitro Study
Source: JMIR Res Protoc. 2026 Sep 8;15:e74309. doi: 10.2196/74309 (PMC13552990; doi:10.2196/74309)
Supplement: Supplementary file 1 [file resprot-v15-e74309-s001.docx]

**Multimedia Appendix 1: Detailed coating parameters, solution recipes, and ICP-OES settings.**

**1. Coating Parameters for Silver Dioxide (Ag₂O/AgO) Thin Film**

**1.1 Substrate Preparation**

- Stainless steel 0.019" × 0.025" archwire segments cut to 30 mm length.
- Surfaces polished using silicon carbide papers in sequence:
  - 800 grit → 1200 grit → 2000 grit (similar to preparation in Zhang et al., *Annals of Biomedical Engineering*, 2014)
- Ultrasonic cleaning in acetone (10 min), followed by ethanol rinse and drying.

**1.2 Deposition Method**

- **RF magnetron sputtering** using a **99.99% pure silver target**.
- Chamber base pressure: **<5 × 10⁻⁵ Torr**
- Working gas: Argon (Ar), with 10% O₂ for oxide formation
- Deposition power: **150 W RF**
- Target–substrate distance: **70 mm**
- Substrate temperature: **room temperature (22–25°C)**
- Deposition time adjusted to achieve **15–20 nm coating thickness**
  - *Thickness corrected based on reviewer comment; mm would be impossible.*
- Sputtering rate: **~0.2 nm/s**

**1.3 Coating Quality Control**

- **Surface morphology**: SEM (ZEISS EVO18)
- **Elemental mapping**: EDS (Oxford INCA X-Max)
- **Phase identification**: XRD (Cu Kα radiation)
- **Chemical structure confirmation**: FTIR used to verify Ag–O bonding
- **Cross-sectional thickness**: Profilometer or FIB-SEM
- **Uniformity checks**: 5% random sample inspection
- **Adhesion test**: Tape test (ASTM D3359)

**2. Solution Recipes Used for Corrosion and Ion Release Studies**

**Solution composition:**

.

**2.1 Chloride Solution (0.9% NaCl)**

- 0.9 g NaCl in 100 mL DI water
- pH: **6.8–7.0**

**2.2 Modified Fusayama Artificial Saliva (AS)**

| **Component** | **Concentration (mg/L)** |
| --- | --- |
| NaCl | 400 |
| KCl | 400 |
| CaCl₂·2H₂O | 795 |
| NaH₂PO₄·H₂O | 690 |
| KSCN | 300 |
| Na₂S·9H₂O | 5 |
| Urea | 1000 |

- Adjust pH to **6.75** using *lactic acid* .
- Filter sterilize (0.22 µm) before use.

**2.3 Fluoride-Containing AS**

- Modified Fusayama AS + **0.1 wt% NaF**

**2.4 Protein-Containing AS**

- Modified Fusayama AS + **0.1% w/v bovine serum albumin (BSA)**
- Gentle stirring required to ensure dissolution.

**2.5 Sterility Considerations**

- All solutions filtered through **0.22 µm membrane**
- Stored at **4°C**
- Used within **7 days**

**3. pH Validation and Control**

Because pH strongly affects corrosion:

- pH measured at **Day 0, Day 14, Day 28** using a calibrated digital pH meter.
- pH adjusted using:
  - **0.1 M lactic acid** (acidifying agent—per parent article).
  - **0.1 M NaOH** (alkalinizing agent).
- Acceptable range maintained within **±0.1 pH units** for experimental consistency.

**4. Immersion Test Parameters**

- Each wire immersed individually in **20 mL** of test solution.
- Duration: **28 days** with mid-point sampling on **Day 14**.
- Incubation:
  - **37°C**
  - **Orbital shaking at 60 rpm** (to simulate salivary flow).
- Fresh solution replaced on Day 14.

**5. ICP–OES Analysis Settings**

Adapted from parent paper (p. 224: ICP-OES using Optima 3300DV; Cu detection limit 0.01 ppm)

generalized for Ag, Ni, Cr, Fe, and Cu.

**5.1 Instrument**

- **PerkinElmer Optima 3300DV** or equivalent dual-view ICP–OES
- Plasma gas: Argon
- RF power: **1300 W**
- Plasma flow rate: **15 L/min**
- Auxiliary gas flow: **0.2 L/min**
- Nebulizer gas flow: **0.8 L/min**

**5.2 Sample Preparation**

- 5 mL immersion solution + 5 mL 2% HNO₃ (trace-metal grade)
- Filter using **0.22 µm PTFE filter**
- Analyze within 24 hours of acidification

**5.3 Elements Measured and Wavelengths**

| **Ion** | **Wavelength (nm)** |
| --- | --- |
| Ag | 328.068 |
| Ni | 231.604 |
| Cr | 267.716 |
| Fe | 238.204 |
| Cu | 324.754 |

**5.4 Calibration**

- Three-point calibration curve: **0.01, 0.1, 1.0 ppm**
- R² ≥ 0.999 required
- Internal standard: **Yttrium (Y) 371 nm**

**5.5 Analytical Sensitivity**

- Detection limit (LOD): **0.01 ppm** for metal ions (as per parent article for Cu)

parent

- Quantification limit (LOQ): **0.05 ppm**

**5.6 Replicates**

- Each sample measured in **triplicate**
- Instrument drift corrected every **10 samples**

**6. SEM/EDS Imaging Parameters**

- Instrument: **ZEISS EVO18 SEM**
- Voltage: **15 kV**
- Working distance: **10 mm**
- Spot size: **3.0**
- EDS detector: Oxford INCA X-Max
- Mapping time: **60–90 seconds per element**
